# TPH2 Gene Polymorphisms and Major Depression – A Meta-Analysis

**DOI:** 10.1371/journal.pone.0036721

**Published:** 2012-05-31

**Authors:** Jin Gao, Zhenglun Pan, Zhian Jiao, Feng Li, Guoqing Zhao, Qianqian Wei, Fang Pan, Evangelos Evangelou

**Affiliations:** 1 Department of Medical Psychology, Shandong University School of Medicine, Jinan, Shandong, China; 2 Department of Clinical Psychology, Provincial Hospital Affiliated to Shandong University, Jinan, Shandong, China; 3 Department of Rheumatology, Provincial Hospital Affiliated to Shandong University, Jinan, Shandong, China; 4 Department of Hygiene and Epidemiology, University of Ioannina School of Medicine, Ioannina, Greece; University of South Florida, United States of America

## Abstract

**Background:**

Tryptophan hydroxylase-2 (TPH2) is the rate-limiting enzyme in the synthetic pathway for brain serotonin and is considered key factor for maintaining normal serotonin transmission in the central neuron system (CNS). Gene-disease association studies have reported a relationship between TPH2 and major depressive disorder (MDD) in different populations, however subsequent studies have produced contradictory results.

**Objectives:**

We performed a systematic overview and a meta-analysis with all available data up-to-date.

**Methods:**

We scrutinized PubMed, Embase, HuGNet and China National Knowledge Infrastructure (CNKI ) and last update was held on October 2011. We also searched the manuscripts and the supplementary documents of the published genome-wide association studies in the field. Effect sizes of independent loci that have been studied in more than 3 articles were synthesized using fixed and random effects models.

**Results:**

We found 27 eligible articles that studied a total of 74 single nucleotide polymorphisms (SNPs). Finally, 12 independent loci were included in the meta-analysis. The synthesis of the data shown that two SNPs (rs4570625 and rs17110747) were associated with MDD using fixed effects models. SNP rs4570625 had low heterogeneity and remained significant using the more conservative random effects calculations with a summary OR = 0.83 (95% CI: 0.73–0.96).

**Conclusion:**

The current study identified a SNP (rs4570625) with strong epidemiological credibility; however more studies are required to provide robust evidence for other weak associations.

## Introduction

Major depressive disorder (MDD) is one of the most common psychiatric disorder [1]. According to the DSM-IV-TR (American Psychiatric Association, 2000), the lifetime risk of major depressive disorder varies from 10 to 25% for women and 5 to 12% for men in community samples. Prevalence rates appear to be unrelated to marital status, education, income, or ethnicity. More than 50% of the individuals who have had a single major depressive disorder episode are expected to have a second episode.

Although the etiology of the disorder has not been well recognized, a genetic component has been found. Twin studies indicate that the heritability of MDD is 0.36 to 0.70 [2]–[7] and the risk of MDD in first-degree relatives of probands is 2 to 4 times larger compared to controls [8]–[12]. It is shown that various genetic factors account for 40–70% of the risk for developing the disorder [13].

Serotonin is one of the main neurotransmitters of the central nervous system (CNS), and it plays an important role in psychiatric disorders. Tryptophan hydroxylase-2 (TPH2) is the rate-limiting enzyme in the synthetic pathway for brain serotonin and is considered key factor for the maintainance of normal serotonin transmission in the CNS [2], [14].

In 2004, Zill et al found two SNPs in TPH2 that were associated with MDD in Caucasians [15]. Subsequently, Zhang and colleagues in 2005 identified the G1463A variant (rs120074175), a rare variant with minor allele frequency <1%, that predicted an amino acid substitution at a highly conserved position in tryptophan hydroxylase2 (TPH2) (p.Arg441His). When expressed in PC12 cells, 441His shown an 80% loss in serotonin production. Genotyping results revealed the presence of the 1463 A-allele in 9 of 87 elderly unipolar affective disorder (UP) patients (>60 years) and in 3 of 219 healthy control individuals [16]. Interestingly, the 1463 A-allele was not replicated in subsequent follow-up studies [17]–[22]. However, the important biological function of TPH2 attracted many researchers to explore a wider range of SNPs covering exons and introns that might identify genetic risk variants that are associated with MDD, with contradictory results though [23], [24]. The current systematic overview aims to identify probable evidence of association of different genetic variants in TPH2 with MDD.

## Materials and Methods

### Search Strategy and Selection Criteria

We scrutinized PubMed, Embase, HuGNet and China National Knowledge Infrastructure (CNKI) using the keywords ‘major depression’, ‘unipolar depression’, ‘major depressive disorder’, ‘MDD’, ‘tryptophanhydroxylase 2′, and ‘TPH2’. Major depression was defined according to the DSM-IV-TR (American Psychiatric Association, 2000), DSM-IIR or ICD10 and is characterized by major depressive episodes without a history of manic, mixed, or hypo manic episodes. A major depressive episode is characterized by a >2 weeks period where there is a new onset or clear worsening of either depressed mood or loss of interest or pleasure in nearly all activities. Four additional symptoms including changes in appetite, weight, sleep, and psychomotor activity; decreased energy; feelings of worthlessness or guilt; difficulty thinking, concentrating, or making decisions; or recurrent thoughts of death or suicidal ideation, plans, or attempts must be also present. The episode must be accompanied by distress or impairment in social, occupational, or other important areas of functioning. (http://omim.org/entry/608516). The data were last accessed on Oct 26, 2011 and we did not set any language restrictions. The references of the retrieved articles were independently screened by two researchers. The eligibility criteria were: a) case- control or cohort studies that quantitatively assessed the relationship of TPH2 gene polymorphism and risk of MDD; b) cases with MDD were eligible regardless of age, gender and ethnicity. Studies without data on allelic counts in cases and controls and studies focusing on survival or other clinical outcome in MDD were excluded from the study. In case of overlapping cases/controls in different studies, we retained only the studies with the largest sample size.

For each study, we recorded the first author, year of publication, the ethnic group of the study population, the method of genotyping, the number of genotyped cases and controls, the source of control population, the standard errors (SE) or the 95% confidence interval (CI), and the odds ratio (OR). Whenever ORs and SEs or 95% CIs were not reported we calculated them from the available data in each paper. We coined the calculated ORs in each study so as to reflect always to the same allelic contrast. Whenever a study included information on a negative strand we used the complimentary allelic strand. We calculated the linkage disequilibrium (LD) for the eligible SNPs using SNAP (http://www.broadinstitute.org/mpg/snap/) for each relevant ethnic population. We considered perfect proxies and therefore included in the same meta-analyses SNPs with r^2^ = 1 and D’ = 1. LD assessement was based on the 1000 genomes panel and a distance limit of 500 mb was set.

### Meta-analysis

For each study we calculated the OR and its respective SE. The heterogeneity of the studies was assessed using the Cochran’s Q test (considered significant for P<0.10) and it was quantified by the I^2^ statistic [25]. I^2^ ranges between 0 to 100% and it is considered low, moderate, large and very large for values 0% −25%, 25% −50%, 50% −75% and >75% respectively. Both fixed effects (Mantel-Haenszel) and random effects (DerSimonian and Laird) models were used to combine the data. Random effects are more conservative incorporating an estimate of the between-study variance and thus provide relatively wider 95% CIs, when heterogeneity exists. We performed a sensitivity analysis on either Caucasians or Asians, based on the larger number of studies for a specific ethnic group. Small vs large studies effect bias was assessed using Harbord’s modified regression test. The analyses were conducted with R package (http://www.R-project.org). All p values were two sided and using Bonferroni correction for the 12 comparisons significance can be deemed at the p = 0.004 level.

## Results

A total of 270 abstracts met the search criteria and were retrieved through Pubmed, EMBASE, HuGNet and CNKI databases. Two reviewers assessed the relevant studies independently. We identified 64 relevant studies that described the association between the TPH2 polymorphism and MDD; however, after scrutinizing the full articles 28 studies were eligible for inclusion in our review and meta–analysis. Specifically 21 out of the 64 identified articles did not meet our inclusion criteria regarding the design of the study and, furthermore, their description of the source of the cases and the controls was inadequate. Moreover, 8 papers included overlapping populations, and 7 papers used dimensional scores rather than diagnoses as an outcome. Among the remaining 28 eligible studies, 1 article did not provide adequate data of TPH2 polymorphisms [26]. Thus, a total of 27 studies were included in our meta-analysis. We also scrutinized the 19 published GWAs studies for reported information; however we were not able to get effect estimates or allele counts for any of our eligible SNPs. Detailed characteristics of all studies included in the meta-analysis are listed in Table S1.

Nineteen studies included participants of European descent, 14 studies included Asians, 3 African Americans and 3 studies participants of other descent (Native Americans and Middle Eastern subjects. All reports selected MDD patients on the basis of DSM-III-R, DSM-IV and all eligible articles pertained case-control studies.

In most of the reports, cases and controls were subsequently randomly selected. Of the eligible studies, 21 described a matched case-control design including participants matched for age and/or gender. 20 reports used hospital controls whereas 7 studies used non-hospital or other types of controls. All cases had clinical diagnosis of MDD at study entry. The detailed methods used for determining the genotypes of TPH2 status are described in each article. Most of the studies included in the present analyses used genomic DNA extracted from blood but one that used brain tissue [27].

Of the 2012 SNPs in TPH2, 74 SNPs across the TPH2 gene area had been assessed and for 5 SNPs we were not able to identify an rsid number. Out of the 69 SNPs that were further considered for the analysis 50 SNPs located in introns; 13SNPs located in exons, 6 SNPs located in 5 UTR. Forty SNPs were reported in less than 3 studies and therefore were excluded from further consideration. The 29 remaining SNPs were locared in 13 independent loci. We excluded rs120074175 because all subsequent studies reported zero counts for the minor allele in cases and controls and therefore we were not able to calculate a robust effect estimate [17]–[22], [28]. Thus 28 SNPs in 12 independent loci were included for the analysis.

**Figure 1:**
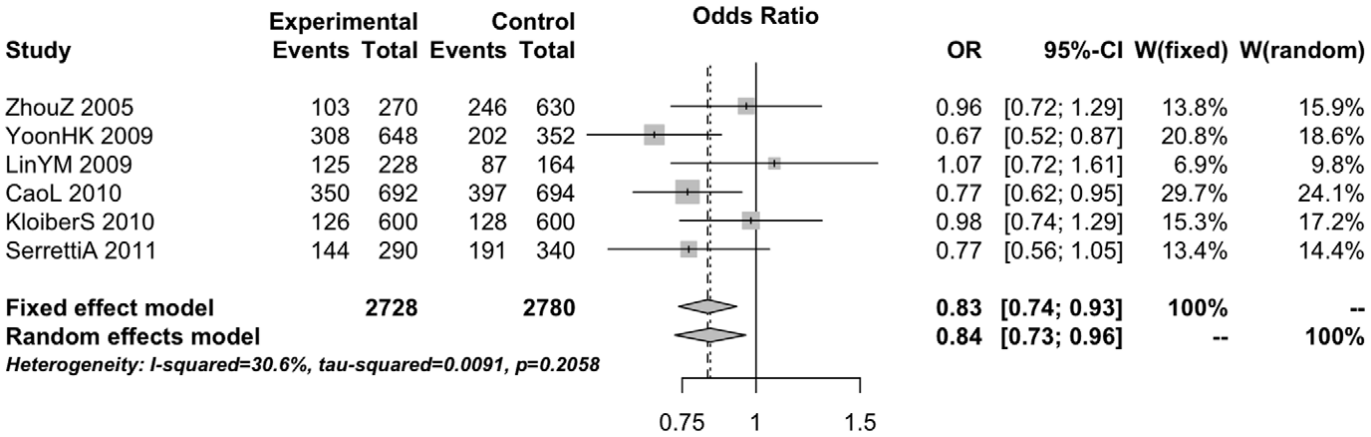
Forest plot of genetic association studies of rs4570625-T and major depression.

Overall, the eligible studies included 13,041 cases and 11,568 controls. For all independent loci, we calculated the summary ORs using fixed and random effects models. The results indicated that rs4570625 was associated with MDD. An analysis of 1364 cases and 1390 controls for rs4570625, including all ethnic groups yielded a summary OR = 0.83 (95%CI: 0.74; 0.93) (p-value = 0.0011), with non-statistically significant between–study heterogeneity (I^2^ = 31%, Cochran’s p-value = 0.21). The random effects summary OR was 0.84 (95% CI: 0.73; 0.96) (Figure 1). A sensitivity analysis including 4 Asian studies shown a summary OR = 0.77 (95% CI: 0.66; 0.90) when random effects were applied.

**Figure 2:**
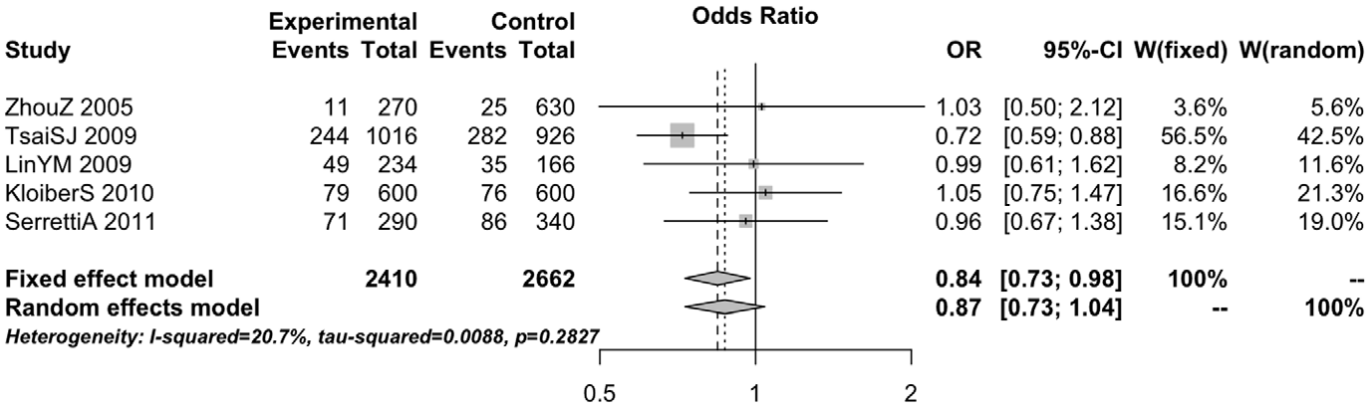
Forest plot of genetic association studies of rs17110747-A and major depression.

The summary OR for rs17110747 (1205 cases and 1331 controls) including all ethnic groups was 0.84 (95% CI: 0.73; 0.98) (p-value = 0.02, with non-statistically significant between–study heterogeneity (I^2^ = 21%, Cochran’s p = 0.28). However the summary OR was not statistical significant when we applied random effects models (Figure 2). Furthermore, the results were similar when only Asian populations were analyzed (Table S2). There is no indication for small vs large study bias in those two findings using the Harbord’s modified regression test with p-values 0.22 and 0.11 respectively.

All the other loci did not yield significant association between the eligible genetic variants and MDD. One other locus (rs11178997) produced a nominally significant result with fixed effects models only, however the association was not present when only Caucasian populations were considered.

## Discussion

We have conducted to the best of our knowledge the first comprehensive systematic overview of genetic studies of TPH2 and MDD. We searched all the candidate-gene studies to identify the eligible SNPs. Furthermore we scrutinized the published genome-wide association studies (GWAs) in the field for additional reported information. We were able to perform meta-analyses of 12 independent loci. We found significant evidence for 2 MDD susceptibility SNPs (rs4570625, rs17110747). It appears that rs4570625 has strong epidemiological credibility. However, we were not able to study most of the SNPs identified due to our exclusion criteria that it was set to three or more studies.

Both identified SNPs shown small to moderate effect, which is well expected in the field of genetic epidemiology. Rs4570625 remained significant after random effect calculations and the total number of cases and controls with the least frequent genetic group exceeds 1000 alleles. Small to moderate heterogeneity was detected and there is no evidence of small-study effect bias and all studies were in HWE. Overall and by applying the Venice interim guidelines [29] that use the amount of evidence the replication of results and the protection of bias for the evaluation of a genetic risk variant indicates that the specific finding could be considered of strong credibility. Rs17110747 was not replicated using random effects models and the evidence should be considered weak even though the observed heterogeneity was small and no small-study effect biases were detected. Interestingly enough this association was stronger in Asian populations but this analysis was limited to only three studies. Therefore more robust inferences are not allowed.

TPH2 was found to be neuronal specific and predominantly expressed in brain serotonergic neurons originating from raphe nuclei. The first publication by Zill et al revealed that rs1386494 and rs1843809 were associated with MDD in Caucasians but we were not able to replicate those findings. However, there is evidence that rs4570625 has an important biological function. Inoue et al found that T allele carriers were associated with significantly smaller volumes in bilateral amygdala and hippocampus and higher reward dependence than those with G allele homozygotes [30]. There are also associations reported with other psychiatric disorders. Zhang et al identified that rs4570625 on TPH2 gene might play an important role in the development of positive symptoms in Han Chinese schizophrenic patients [31] and Kim et al reported that the specific polymorphism may has a significant contribution in the pathogenesis of panic disorder [32]. More recently, Leppanen et al found infants with the T-carrier genotype exhibited a significantly higher number of missing attention shifts [33]. Regarding rs17110747, there is no significant evidence that the specific risk variant is a loss-of-function mutation and further studies are needed to support such a finding.

Our study has several limitations. Although we have tested for small-study effects, publication biases cannot be entirely excluded from retrospective meta-analysis, regardless our extensive search in different databases. Also, for many meta-analyses the total number of cases and controls studied was not large enough and therefore we are underpowered to detect even moderate associations (OR = 1.2–1.5). Moreover, we were able to synthesize results from only 2/5 of the identified studies, thus we did not benefit from all the available information. We excluded studies where the genotypic distribution in controls was not in Hardy-Weinberg equilibrium or studies that did not clearly distinguish between MDD and bipolar disease. As a consequence, there were 46 polymorphisms that were only addressed in one or two eligible studies. However, by adopting the criterion of three or more studies gave us the opportunity to diminish possible false positive signals that could derive from the synthesis of a small amount of information. Last but not least, we searched only the manuscript and the supplementary documents of the published GWAs and therefore we would not be able to detect any of our eligible SNPs that did not reached pre-specified levels of significance.

Moreover, we did not exclude studies where the controls were recruited based on different criteria. For example, we did not distinguish between controls that had been screened for the absence of depression and controls that were selected from the general population or the hospital. Such differences may significantly contribute to the heterogeneity observed in several associations. It would be essential that individual studies report key characteristics of their populations that would allow for the assessment of the observed heterogeneity and the evaluation of possible misclassification in future efforts. To provide robust and strong epidemiologic evidence in gene-disease association studies, harmonization of the study designs, populations and measurements is of great importance and it one of the aims suggested by the Network of Investigator Networks of the Human Genome Epidemiology Network [34].

Our systematic meta-analysis found strong epidemiologic credibility for rs4570625 and significant evidence but weaker credibility for rs17110747 in TPH2. The low coverage of genetic variants of TPH2 makes it impossible to conclude that the other SNPs studied are not involved in MDD. Efforts are required to standardize the methodology in research of MDD and TPH2 gene polymorphisms and further research aiming to replicate findings in MDD is needed.

## Supporting Information

Table S1 **Summary of stydies of major depression and TPH2 polymorphisms** [**15]**–[24], [26]–[28], [35]–[50****] **.**
(PDF)

Table S2 **Meta-analyses of genetic association studies of TPH2 gene polymorphisms and major depression.**
(PDF)
