# Supplementary material for: TPH2 Gene Polymorphisms and Major Depression – A Meta-Analysis
Source: PLoS One. 2012 May 31;7(5):e36721. doi: 10.1371/journal.pone.0036721 (PMC3365065; doi:10.1371/journal.pone.0036721)
Supplement: Table S1 — Summary of stydies of major depression and TPH2 polymorphisms [15] –[24], [26]–[28], [35]–[50] . (PDF) [file pone.0036721.s001.pdf]

Table 1. Summary of studies of MDD and TPH2 polymorphisms

Year	Investigator	Place of study	Selection/characteristics of cases and controls		Race	Eligible subjects		Source of controls	Method
			cases	controls		cases	controls		
2004	Zill P[15]	Germany	DSM-IV / ICD-10	MMPI/NEO-FI-TCI/short structured interview with a psychiatrist	Caucasian	300	265	ethnically matched from the general population in Southern Germany	sequencing
2005	Zhou ZF[22]	U.S.	DSMIII-R/ DSM-IV	NA	Caucasian/African American/American Indian/Hispanic/Asian -American	2143	352	NA	sequencing and genotyping by 5'-nuclease assay
2005	Van Den Bogaert A[21]	Sweden/ Belgium	DSM-IV	NA	Caucasian	681	728	healthy age-, gender, and ethnicity-matched control individuals	sequencing
2005	Garriock HA [19]	U.S.	DSM-III-R	without personal history of major affective disorders	Caucasian/Asian、Hispanic/African American/Native American/Middle Eastern	190	186	NA	sequencing
2005	Glatt CE[20]	U.S.	DSM-IV	NA	European/American/Hispanic/ African American/Asian and other	1023	0	None	genotyping by 5'-nuclease assay
2005	Zhang XD[16]	U.S.	DSM-IV	NA	Caucasian	147	219	NA	ARMS-PCR
2005	Zhou ZF[24]	U.S.	DSM-III-R/DSM-IV	free of psychiatric disorders	US whites/Finnish whites/African Americans/Southwestern American Indians	190	1798	healthy age-, gender-, and ethnicity-matched control individuals	sequencing and genotyping by 5'-nuclease assay
2006	Van Den Bogaert A[35]	Switzerland .	DSM-IV	NA	Swidish	135	364	ethnicity-matched control individuals	pyrosequencing

2006	Bicalho MA[17]	Brazil	DSM-IV /the Geriatric Depression Scale	without personal or familial history of psychiatric disorders	Caucasian	102	57	NA	PCR sequencing
2006	Delorme R[18]	Switzerland.	DSM-IV	Absence of personal and familial history of psychiatric disorders	Caucasian/Asian	1071	523	ethnicity-matched control individuals	ARMS-PCR PCR-RFLP,sequencing
2008	Jiang WY[36]	China	DSM-IV/ HAMD	interview with a psychiatrist, free of psychiatric disorders	Asian	170	222	ethnically matched subjects from the general population in health volunteer	PCR－RFLP
2008	Haghighi F[27]	U.S.	DSM-IV/SCID-I / SCID-II;	non-major depression and non-suicide	Caucasians	82	80	NA	PCR、sequencing、
2008	Mann JJ[37]	U.S.	DSM-IV/ SCID-I/ SCID-II/SCID-NP	SCID-NP	Caucasians/ Asian	324	130	healthy volunteers responded to advertisements	PCR-RFLP
2008	Gizatullin R[28]	Sweden	DSM-III-R / DSM-IV	free from any psychiatric illness	Caucasians	194	246	age,ethnicity,and geographical distribution matched.	PCR-RFLP
2009	Illi A[38]	Finland	DSM-IV/	NA	Caucasians	192	395	healthy Finnish blood donors. The mean age of the control group was higher than that of the study group	real-time PCR
2009	Yoon HK[39]	Korea	DSM-IV/ HDRS	SCID-NP	Asian	324	176	gender, age and education matched, without medical/psychiatric illness nor family history of mental illness in first- and second-degree	PCR

								relatives.	
2009	Tsai SJ[40]	China	DSM-IV/ HAMD	psychiatric interview to rule out past or present psychosis, mood disorders, et al.	Asian	508	403	community volunteers or medical staff	PCR-SSP
2009	Utge S[41]	Finland	DSM-IV	with no depression or any other psychiatric disorder according to the CIDI interview	Caucasians	1654	1270	NA	PCR-SSP
2009	Lin YM[42]	China	DSM-IV	with no history of depression or anxiety	Asian	117	83	age-matched	real-time PCR, sequencing
2010	Xue KX[43]	China	DSM-IV/CCMD-3/HAMD	absence of personal and familial history of psychiatric disorders	Asian	123	122	community volunteers or medical staff	PCR、ARMS-PCR、RFLP
2010	Kloiber S[23]	Germany	DSM-IV	screened for the presence of anxiety and affective disorders using the Composite International Diagnostic Screener	Caucasians	988	1023	ethnicity, sex and age matched, randomly selected from a Munich-based community sample	MassARRAY
2010	Cao J[44]	China	ICD-10/DSM-IV/HAMD	absence of personal and familial history of psychiatric disorders	Asian	102	102	ethnicity, sex and age matched ,community volunteers	PCR－RFLP
2010	Cao L[45]	China	DSM-IV	absence of personal and familial history of psychiatric disorders	Asian	346	347	ethnicity, sex and age matched ,community volunteers	PCR
2011	Serretti A[46]	Korea	DSM-IV/MADRS/YM	psychiatrically healthy	Asian	498	170	ethnicity matched, community volunteers	pyrosequencing assay

			RS						
2011	Pereira Pde A[47]	Brazil	DSM-IV	no personal and family history in first degree relatives of neuropsychiatry diseases, including no alcohol abuse	Caucasians	84	79	selected among patients followed in the Clinic	real-time PCR
2011	Soronen P[26]	Finland	DSM-IV/SCID-I/P/JoBS/ PC-VDS	with no depression or any other psychiatric disorder according to the Composite International Diagnostic Interview	Caucasians	450	1322	the population-based Health 2000 study of Finland	MassARRAY
2011	Claes S[48]	Belgium	DSM-IV/SCID-I/ HAM-D-21	NA	Caucasians	338	310	gender, age matched	MassARRAY
2011	Zhang YQ[49]	China	CCMD-3/ HAMD	no personal and family history in first degree relatives of neuropsychiatry diseases	Asian	197	225	ethnicity, sex and age matched ,community volunteers	sequencing
2011	Shen XH[50]	China	DSM-IV/HAMD	Structural Clinical Interview for DSM-IV Disorders (SCID). psychiatrically healthy	Asian	368	371	ethnicity, sex and age matched ,community volunteers	PCR-RFLP
