# Supplementary material for: TPH2 Gene Polymorphisms and Major Depression – A Meta-Analysis
Source: PLoS One. 2012 May 31;7(5):e36721. doi: 10.1371/journal.pone.0036721 (PMC3365065; doi:10.1371/journal.pone.0036721)
Supplement: Table S2 — Meta-analyses of genetic association studies of TPH2 gene polymorphisms and major depression. (PDF) [file pone.0036721.s002.pdf]

**Table 2 Meta-analyses of genetic association studies of TPH2 gene polymorphism and major depression**

| SNPs | studies | ethnicity | OR | Fixed (95% CI) | pvalue | OR | Random (95% CI) | pvalue | $I^2$ | Harbord's test p |
|---|---|---|---|---|---|---|---|---|---|---|
| rs1386492-G | 4 | all | 0.8758 | 0.7642; 1.0037 | 0.0566 | 0.8758 | 0.7642; 1.0037 | 0.0566 | 0% [0%; 67%] | 0.5417 |
| | 3 | Caucasian | 0.9001 | 0.7593; 1.067 | 0.2253 | 0.9001 | 0.7593; 1.0670 | 0.2253 | 0% [0%; 81%] | 0.9259 |
| rs1386494-A | 9 | all | 0.9185 | 0.8026; 1.0512 | 0.217 | 0.9435 | 0.7359; 1.2098 | 0.6467 | 66% [31%; 83%] | 0.7353 |
| rs1386495-C | | | | | | | | | | |
| rs1843809-G | 7 | Caucasian | 0.9023 | 0.7853; 1.0367 | 0.1467 | 0.9182 | 0.7384; 1.1416 | 0.4424 | 56% [0%; 81%] | 0.6858 |
| rs11179000-T | 3 | Caucasian | 1.0293 | 0.8559; 1.2379 | 0.7587 | 1.0262 | 0.8447; 1.2467 | 0.7947 | 7% [0%; 90%] | 0.4836 |
| rs17110690-A | | | | | | | | | | |
| rs6582072-A | 3 | Caucasian | 0.0849 | 0.6772; 1.0254 | 0.8333 | 0.6953 | 0.4108; 1.1767 | 0.1758 | 81% [41%; 94%] | 0.1991 |
| rs1386493-A | | | | | | | | | | |
| rs2129575-T | 4 | Caucasian | 0.9440 | 0.8044; 1.1079 | 0.4806 | 0.8948 | 0.6790; 1.1792 | 0.4299 | 64% [0%; 88%] | 0.04221 |
| rs4131348- C | 3 | Caucasian | 0.8664 | 0.7497; 1.0014 | 0.0522 | 0.8680 | 0.7486; 1.0064 | 0.0608 | 3% [0%; 90%] | 0.3891 |
| rs17110467- A | | /Asian | | | | | | | | |
| rs17110451-G | | | | | | | | | | |
| rs4290270-T | 7 | all | 1.0903 | 0.9969; 1.1925 | 0.0585 | 1.0870 | 0.9815; 1.2039 | 0.1094 | 21% [0%; 64%] | 0.7353 |
| rs4474484-G | | | | | | | | | | |
| rs4469933-T | | | | | | | | | | |
| rs2200579-A | | | | | | | | | | |
| rs1386486-G | 4 | Caucasian | 1.0155 | 0.8921; 1.156 | 0.8158 | 1.0155 | 0.8921; 1.1560 | 0.8158 | 0% [0%; 84%] | 0.8655 |

| | | | | | | | | | | |
|---|---|---|---|---|---|---|---|---|---|---|
| rs4570625-T | 6 | all | 0.8283 | 0.7396; 0.9277 | 0.0011 | 0.8366 | 0.7283; 0.9611 | 0.0117 | 31% [0%; 72%] | 0.2221 |
| | 4 | Asian | 0.7692 | 0.6706; 0.8822 | 0.0002 | 0.7734 | 0.6626; 0.9027 | 0.0011 | 18% [0%; 87%] | 0.3221 |
| rs7305115-G<br>rs2171363-C<br>rs7979770-T<br>rs6582078-G<br>rs4760815-T | 9 | all | 0.9321 | 0.8638; 1.0058 | 0.07 | 0.9321 | 0.8638; 1.0058 | 0.07 | 0% [0%; 42%] | 0.5473 |
| | 5 | Caucasian | 0.9622 | 0.8612; 1.0751 | 0.4961 | 0.9622 | 0.8612; 1.0751 | 0.4961 | 0% [0%; 77%] | 0.6813 |
| rs10748185-A<br>rs10784941-G | 6 | all | 1.0859 | 0.9741; 1.2105 | 0.137 | 1.1238 | 0.9365; 1.3486 | 0.2096 | 63% [11%; 85%] | 0.02069 |
| | 5 | Caucasian | 1.0567 | 0.9412; 1.1863 | 0.3503 | 1.0921 | 0.8923; 1.3368 | 0.3927 | 66% [12%; 87%] | 0.06091 |
| rs11178997-A<br>rs11178998-G | 4 | all | 0.7538 | 0.4014; 1.0611 | 0.0136 | 0.6526 | 0.6022; 0.9436 | 0.0853 | 74% [27%; 91%] | 0.1932 |
| | 3 | Caucasian | 0.8804 | 0.6804; 1.1392 - | 0.3328 | 0.7699 | 0.4624; 1.2817 | 0.3145 | 65% [0%; 90%] | 0.2046 |
| rs17110747-A | 5 | all | 0.8442 | 0.7300; 0.9763 | 0.0224 | 0.8722 | 0.7305; 1.0414 | 0.1308 | 21% [0%; 66%] | 0.1096 |
| | 3 | Asian | 0.7938 | 0.6729; 0.9364 | 0.0062 | 0.8193 | 0.6625; 1.0133 | 0.066 | 26% [0%; 92%] | 0.1735 |
